# Design, Synthesis and Biological Evaluation of4-(Imidazolylmethyl)-2-(4-methylsulfonyl phenyl)-Quinoline Derivatives as Selective COX-2 Inhibitors and *In-vitro *Anti-breast Cancer Agents

**Published:** 2016

**Authors:** Razieh Ghodsi, Ebrahim Azizi, Afshin Zarghi

**Affiliations:** a *Biotechnology Research Center, Mashhad University of Medical Sciences, Mashhad, Iran. *; b *Department of Toxicology, School of Pharmacy, Tehran University of Medical sciences, Tehran, Iran.*; c *Department of Pharmaceutical Chemistry, School of Pharmacy, Shahid Beheshti University of Medical Sciences, Tehran, Iran.*

**Keywords:** Quinolines, COX-2 inhibitory, Aromatase inhibitory, Breast cancer

## Abstract

A new group of 4-(Imidazolylmethyl**)**quinoline derivatives possessing a methylsulfonyl COX-2 pharmacophore at the *para* position of the C-2 phenyl ring were designed and synthesized as selective COX-2 inhibitors and *in-vitro*anti breast cancer agents.

*In-vitro* COX-1 and COX-2 inhibition studies showed that all the compounds were potent and selective inhibitors of the COX-2 isozyme with IC_50_ values in the potent range 0.063-0.090 µM, and COX-2 selectivity indexes in the 179.9 to 547.6 range. Molecular modeling studies indicated that the methylsulfonyl substituent can be inserted into the secondary pocket of COX-2 active site for interactions with Arg^513^. Cytotoxicity of quinolines 9a-e against human breast cancer MCF-7 and T47D cell lines were also evaluated. All the compounds 9a-e were more cytotoxic against MCF-7 cells in comparison with those of T47D which express aromatase mRNA less than MCF-7 cells.The data showed that the increase of lipophilic properties of substituents on the C-7 and C-8 quinoline ring increased their cytotoxicity on MCF-7cells andCOX-2 inhibitory activity. Among the quinolines 9a-e, 4-((1*H*-Imidazol-1-yl)methyl) 7,8,9,10-tetrahydro-2-(4-methylsulfonylphenyl)-benzo[h]quinoline (9d)was identified as the most potent andselective COX-2inhibitor as well as the most cytotoxic agent against MCF-7 cells.

## Introduction

Breast cancer is one of the leading causes of cancer-related mortality among women worldwide (1). In most of cases, it is hormone-dependent because tumor progression is dependent on high levels of circulating estrogens, which help the cancer cells to proliferate. Moreover, in postmenopausal women, biologically active estrogens are locally produced from circulating inactive steroids in an intracrine mechanism in breast cancer tissues and confer estrogenic activities to carcinoma cells (2) A series of enzymes are involved in this intratumoral or in situ production of estrogens in breast carcinoma tissues, aromatase, a member of the cytochrome P450 family, is the key enzyme in this process, promoting the aromatization of theAring of androgen precursors (3) the other enzyme involved in breast cancer are COX-2.In addition to the role of COX-2 in inflammatory disorders such as rheumatoid arthritis and osteoarthritis, it is also implicated in cancer and angiogenesis. In this regard, several epidemiologic studies have been reported that inhibitors of COX-2 enzyme reduce the risk of colorectal, breast, and lung cancer, and COX-2 is expressed in these cancers(4-6). So we decided to design quinoline derivatives as dual inhibitors of COX-2 and aromatase to find new approaches for the prevention and treatment of breast cancer.

**Figure 1:**
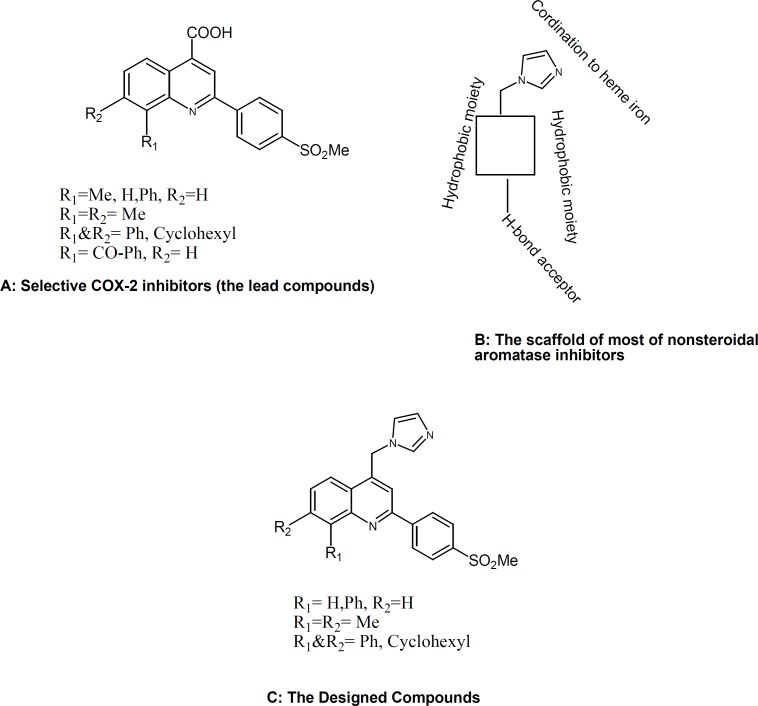
Chemical structures of our lead compounds (A and B) and our designed compounds (C).

We recently reported several investigations describing the design, synthesis, and a molecular modeling study for a group of 2-phenyl-4-carboxyl quinolones (Figure 1, A) possessing a methyl sulfonyl COX-2 pharmacophore at the *para* position C-2 phenyl ring (7, 8).Our results showed that quinoline ring is a suitable scaffold for COX-2 inhibitory activity. On the other hand, the structure of nonsteroidal aromatase inhibitors can be considered to consist of two parts. One part is the azole part with a nitrogen atom which interacts with the heme iron atom of the cytochrome P450 of aromatase. The second part is the bulky aryl part, which mimics the steroid ring of the substrate (andrestendione) (Figure1, B)(8, 9).

So we changed the carboxyl group of our selective COX-2 inhibitors (possessing a methyl sulfonyl COX-2 pharmacophore at the *para* position C-2 phenyl ring) with the imidazolering, the main pharmacophore for anti-aromatase activityn(9) and as shown in Fig. 1, our designed compounds (C)are possessing bulky aryl part, which are important for inhibiting both aromatase and COX-2 enzymes.

## Experimental

Chemistry

All chemicals and solvents used in this study were purchased from Merck AG and Aldrich Chemical. Melting points were determined with a Thomas-Hoover capillary apparatus. Infrared spectra were acquired using a Perkin Elmer Model 1420 spectrometer. A Bruker FT-500 MHz instrument (Brucker Biosciences, USA) was used to acquire 1H NMR spectra with TMS as internal standard. Chloroform-D and DMSO-d6 were used as solvents. Coupling constant (J) values are estimated in hertz (Hz) and spin multiples are given as s (singlet), d (double), t (triplet), q (quartet), m (multiplet), and br (broad). The mass spectral measurements were performed on an 6410Agilent LCMS triple quadrupole mass spectrometer(LCMS) with an electrospray ionization (ESI) interface. Microanalyses, determined for C and H, were within ± 0.4% of theoretical values.

*General procedure for preparation of 2-(4-(methylthio)phenyl)-7,8-substituted-quinoline-4-carboxylic acid (4a-4e)*

These compounds were synthesized according to our pervious methods(7, 11).

*General procedure for preparation of 2-(4-methylthio-phenyl)-7,8-substituted-quinoline-4-yl)methanol (7a-7e)*

LiAlH_4 _(0.45 g, 12 mmol) was suspended in dry THF (20 ml) under a nitrogen atmosphere. Under vigorous stirring a solution of appropriate acid(4a-4e) (5.67 mmol) in dry THF was added dropwise to keep the reaction mixture slightly boiling. After stirring for 2 h at room temperature, the suspension was carefully hydrolyzed with NaOH solution (10%) till no more hydrogen was produced. The solid was filtered off and washed thoroughly with chloroform(12). The filtrate was dried over Na_2_SO_4 _and the solvent was removed under reduced pressure to yield a yellow oil which was crystallized from ether/hexane (25:75 v/v) (yield: 62-79 %).

*2-(4-Methylthio-phenyl)-8-phenylquinoline-4-yl)methanol (7a)*

Yield: 77%; yellow crystalline powder; mp=131-133˚C; IR (KBr): ν (cm^-1^), 3745-2371(OH);LCMS (ESI): 380.6 (M+23)^ +^100.

*2-(4-Methylthio-phenyl)-7,8-dimethylquinoline-4-yl)methanol (7b)*

Yield: 62%; pale yellow crystalline powder; mp=140-141˚C; IR (KBr): ν (cm^-1^), 3406(OH);

LCMS (ESI): 310.2 (M+1)^ +^100.

*2-(4-Methylthio-phenyl)-benzo[h]quinoline-4-yl)methanol (7c)*

Yield: 68%; yellow crystalline powder; mp=111-113˚C; IR (KBr): ν (cm^-1^), 3290(OH);

LCMS (ESI): 332.9 (M+1)^ +^100.

*7,8,9,10-Tetrahydro-2-(4-methylthio-phenyl)-benzo[h]quinoline-4-yl)methanol (7d)*

Yield: 79%; pale yellow crystalline powder; mp=139-140^º^C; IR (KBr): ν (cm^-1^), 3234(OH);

LCMS (ESI): 336.9 (M+1)^ +^100.

*2-(4-Methylthio)phenyl- quinoline-4-yl)methanol (7e)*

Yield: 63%; yellow crystalline powder; mp=120-122^º^C; IR (KBr): ν (cm^-1^), 3224(OH);

LCMS (ESI): 282.1 (M+1)^ +^100.

*General procedure for preparation of 2-(4-methylsulfonyl-phenyl)-7,8-substituted-quinoline-4-yl)methanol (8a-8e)*

One gram of 2-(4-methylthio)phenyl-7,8-substituted-quinoline-4-yl)methanol (7a-7e)was dissolved in 10 ml THF and 5 g oxone in THF/water was added. The mixture was stirred at room temperature for 3-5 h, after evaporation of THF, the residue was extracted with chloroform and dried with sodium sulfate and then evaporated(13), the product was recrystallized in chloroform/hexane (yields: 67-70%).

*2-(4-Methylsulfonyl-phenyl)-8-phenylquinoline-4-yl)methanol (8a)*

Yield: 48%; yellow crystalline powder; mp=178-179^º^C; IR (KBr): ν (cm^-1^), 3442(OH) 1312, 1157(SO_2_);LCMS (ESI): 390.8 (M+1)^ +^100.

*2-(4-Methylsulfonyl-phenyl)-7,8-dimethylquinoline-4-yl)methanol (8b)*

Yield42%; orange crystalline powder; mp=148-150ºC; IR (KBr): ν (cm^-1^), 3241(OH), 1303, 1149(SO_2_);LCMS (ESI): 342.1(M+1)^ +^100.

*2-(4-Methylsulfonyl-phenyl)-benzo[h]quinoline-4-yl)methanol (8c)*

Yield: 65%; yellow crystalline powder; mp=210-211ºC; IR (KBr): ν (cm^-1^), 3485 (OH), 1296, 1145(SO_2_);^1^HNMR (DMSO-d_6 _): δ(ppm) 3.28(s, 3H, SO_2_Me), 5.18 (s, 2H, CH_2_), 5.71 (s, 1H, OH), 7.74-7.80 (m, 2H, benzoquinoline H_8_&H_9_), 7.96-7.80(m, 2H,benzoquinoline H_6_&H_5_), 7.99 (d, 1H,benzoquinoline H_7_) 8.11 (d, 2H, 4-methylsulfonylphenyl H_2_&H_6_, J=8.46Hz), 8.37 (s, 1H,benzoquinoline H_3_), 8.63 (d, 2H, 4-methylsulfonylphenyl H_3_&H_5_, J=8.46Hz), 9.4 (d, 1H, quinoline H_10_, J=7.34Hz); LCMS(ESI): 364.1 (M+1)^+^100.

*7,8,9,10-Tetrahydro-2-(4-methylsulfonyl-phenyl)-benzo[h]quinoline-4-yl)methanol (8d)*

Yield: 57%; yellow crystalline powder; mp=162-163ºC; IR (KBr): ν (cm^-1^), 3365 (OH), 1309, 1159(SO_2_);LCMS (ESI): 368.9 (M+1)^ +^100.

*2-(4-Methylsulfonyl)phenyl-quinoline-4-yl)methanol (8e)*

Yield: 75%; yellow crystalline powder; mp=164-166ºC IR (KBr): ν (cm^-1^), 3340(OH), 1301, 1151(SO_2_);LCMS (ESI): 314.9 (M+1)^ +^100.

*General procedure for preparation of 4-((1H-imidazol-1-yl)methyl)-2-(4-methylsulfonyl-phenyl)-7,8-substituted-quinoline*

To a solution of the corresponding alcohol (1.59 mmol) in 15 ml NMP (N-methyl-2-pyrrolidone), CDI (carbonyl 1, 1-diimidazole) (1.29 g, 7.9 mmol) was added. Then the solution was heated to reflux for 20 h at 170 ºC. After cooling to ambient temperature, it was diluted with water (50 ml) and extracted with ethyl acetate. The combined organic phases was washed with brine and water, dried over Na_2_SO_4,_ and evaporated under reduced pressure (14). Then the desired product was purified by flash chromatography on silica gel (dichloromethane/methanol, 90:10 v/v), (yield: 13-62%).

*4-((1H-Imidazol-1-yl)methyl)-2-(4-methylsulfonyl phenyl)-8-phenyl-quinoline (9a)*

Yield: 25%; cream crystalline powder; mp=196-198ºC;IR (KBr): ν (cm^-1^), 1310, 1150 (SO_2_);^1^HNMR (CDCl_3 _):δ(ppm) 3.03 (s, 3H, SO_2_Me) 5.73 (s, 2H, CH_2_), 7.04 (s, 1H, imidazole H_5_), 7.23(s, 1H, imidazole H_2_), 7.34(s, 1H, imidazole H_4_), 7.45 (t, 1H, phenyl H_4_, J=7.15Hz), 7.52 (t, 2H, phenyl H_3_&H_5_, J=7.31Hz), 7.69-7.76(m, 4H, quinoline H_6_&H_7_&phenyl H_2 _&H_6_), 7.86 (d, 1H, quinoline H_5,_ J=6.88 Hz ),7.92 (s, 1H, quinoline H_3_), 7.95 (d, 2H, 4-methoxysulfonylphenyl H_2_&H_6_, J=8.13Hz), 8.14 (d, 2H, 4-methoxysulfonylphenyl H_3_&H_5_, J=8.13Hz); LCMS(ESI): 440.6 (M+1)^+^100.

*4-((1H-Imidazol-1-yl)methyl)-2-(4- methylsulfonyl phenyl)-7,8-dimethyl-quinoline (9b)*

Yield: 61%; yellow crystalline powder; mp=218-219ºC; IR (KBr): ν (cm^-1^), 1301, 1150 (SO_2_); ^1^HNMR (CDCl_3 _): δ(ppm) 2.58(s, 3H, Me), 2.91(s, 3H, Me), 3.12(s, 3H, SO_2_Me), 5.76 (s, 2H, CH_2_), 7.05 (s, 1H, imidazole H_5_), 7.26(s, 1H, imidazole H_4_), 7.39(s, 1H, imidazole H_2_), 7.50 (d, 1H, quinoline H_6_, J=8.53Hz), 7.73 (d, 1H, quinoline H_5_,J=8.53Hz), 8.01 (s, 1H, quinoline H_3_), 8.08 (d, 2H, 4-methylsulfonylphenyl H_2_&H_6_, J=8.53Hz), 8.26 (d, 2H, 4-methylsulfonylphenyl H_3_&H_5_, J=8.53Hz); LCMS(ESI): 392.1 (M+1)^+^100.

*4-((1H-Imidazol-1-yl)methyl)-2-(4- methylsulfonyl phenyl)-benzo[h]-quinoline (9c)*

Yield: 25%; cream crystalline powder; mp=216-217ºC; IR (KBr): ν (cm^-1^) 1303, 1148 (SO_2_);^1^HNMR (DMSO-d_6 _): δ(ppm) 3.26(s, 3H, SO_2_Me), 5.90 (s, 2H, CH_2 _), 7.01 (s, 1H, imidazole H_5_), 7.36(s, 1H, imidazole H_2_), 7.79 (m, 2H, benzoquinoline H_8_&H_9_), 7.93(s, 1H, imidazole H_4_), 8.03-8.05(m, 3H,benzoquinoline H_3_&H_6_&H_7_), 8.10 (d, 2H, 4-methylsulfonylphenyl H_2_&H_6_, J=8.32Hz), 8.14 (d, 1H,benzoquinoline H_10_), 8.50 (d, 2H, 4-methylsulfonylphenyl H_3_&H_5_, J=8.32Hz), 9.4 (d, 1H, quinoline H_5_); LCMS(ESI): 414.6 (M+1)^+^100.

*4-((1H-Imidazol-1-yl)methyl)-7,8,9,10-tetrahydro-2-(4-methylsulfonylphenyl)-benzo[h]-quinoline (9d)*

Yield: 35%; cream crystalline powder; mp=247-248ºC; IR (KBr): ν (cm^-1^) 1318, 1163(SO_2_);^1^HNMR (CDCl_3 _): δ(ppm) 1.9-2.01(m, 4H, CH_2_), 2.96 (m, 2H, CH_2_), 3.08 (s, 3H, SO_2_Me), 3.46 (m, 2H, CH_2_),5.67 (s, 2H, CH_2 _), 7.0 (s, 1H, imidazole H_5 _), 7.2(s, 1H, imidazole H_2 _), 7.29 (s, 1H, imidazole H_4 _), 7.35 (d, 1H, quinoline H_6_ J=8.57 Hz), 7.66 (d, 1H, quinoline H_5,_ J=8.58 Hz), 7.70 (s, 1H, quinoline H_3_), 8.03 (d, 2H, 4-methoxysulfonylphenyl H_2_&H_6_, J=8.46Hz), 8.29 (d, 2H, 4-methoxysulfonylphenyl H_3_&H_5_, J=8.46Hz); LCMS(ESI): 418.7 (M+1)^+^100.

*4-((1H-Imidazol-1-yl)methyl)-2-(4- methylsulfonyl phenyl)-quinoline (9e)*

Yield: 15%; cream crystalline powder; mp=189-190ºC; IR (KBr): ν (cm^-1^) 1300, 1150(SO_2_);^1^HNMR (CDCl_3 _): δ (ppm) 3.1(s, 3H, SO_2_Me), 5.76 (s, 2H, CH_2_), 7.07 (s, 1H, imidazole H_5_), 7.26(s, 1H, imidazole H_4_), 7.29(s, 1H, imidazole H_2_), 7.69-7.72 (m, 2H, quinoline H_6_&H_3_), 7.87 (t, 1H, quinoline H_7_, J=7.27Hz), 7.98 (d, 1H, quinoline H_5_, J=8.28Hz), 8.09 (d, 2H, 4-methylsulfonylphenyl H_2_&H_6_, J=8.37Hz), 8.26 (d, 2H, 4-methylsulfonylphenyl H_3_&H_5_, J=8.37Hz), 8.3 (d, 1H, quinoline H_8_, J=8.40Hz); LCMS(ESI): 364.8 (M+1)^+^100.

*Molecular modeling (docking) studies*

Docking studies were performed using Autodock software Version 3.0. The coordinates of the X-ray crystal structure of the selective COX-2 inhibitor SC-558 bound to the murine COX-2 enzyme was obtained from the RCSB Protein Data Bank (1cx2) and hydrogens were added. The ligand molecules were constructed using the Builder module and were energy minimized for 1000 iterations reaching a convergence of 0.01 kcal/mol.The energy minimized ligands were superimposed on SC-558 in the PDB file 1cx2 after which SC-558 was deleted. The purpose of docking is to search for favorable binding configuration between the small flexible ligands and the rigid protein. Protein residues with atoms greater than 7.5 A from the docking box were removed for efficiency. These docked structures were very similar to the minimized structures obtained initially. The quality of the docked structures was evaluated by measuring the intermolecular energy of the ligand–enzyme assembly (15, 16).

*In-vitro cyclooxygenase (COX) inhibition assays*

The assay was performed using an enzyme chemiluminescentkit (Cayman Chemical, MI, USA) according to our previously reportedmethod(17)The Cayman chemical chemiluminescent COX

(ovine) inhibitor screening assay utilizes the heme-catalyzedhydroperoxidase activity of ovine cyclooxygenases to generateluminescence in the presence of a cyclic naphthalene hydrazideand the substrate arachidonic acid. Arachidonate-induced luminescencewas shown to be an index of real-time catalytic activity anddemonstrated the turnover inactivation of the enzyme. Inhibition

of COX activity, measured by luminescence, by a variety of selectiveand non-selective nhibitors showed potencies similar to those observedwith other *in-vitro* and whole cell methods.

*Cytotoxicity assay*

*Cell line and culture conditions*

The human breast cancer T47D and MCF-7 cell lines were obtained from Pasteur Institute Cell Bank of IRAN (Tehran, IRAN). Cells were maintained in RPMI-1640(Gibco, UK) culture medium supplemented with 10% fetal bovine serum (Gibco, UK) and 100 U Ml^-1^ of penicillin and 100 ng Ml^-1^ of streptomycin (Gibco, UK) at 37 C in 5% CO_2_ incubator. All reagent and chemicals used in this experiment were of cell culture or molecular biology grade, purchased from different international sources.

*General procedure*

The MTT (3-[4, 5-dimethylthiazol-2-yl]-2,5-diphenyl tetrazolium bromide) based assay was performed by seeding 5000 cells (T47D and MCF-7) per 180 µL RPMI complete culture medium in each well of 96-well culture plates. The day after seeding, culture medium was changed with medium containing standard anti-tumor drug Doxorubicin as well as different concentrations of newly synthesized compounds and RPMI control (no drug). Cells were then incubated at 37ºC in 5% Co2 incubator for 48h and 72h. Then 25 µL of MTT solution (4mg Ml^-1^) were added to each well and further incubated at 37ºC for 3h. At the end of incubation, formazan crystals were dissolved in 100 µL of DMSO and plates were read in a plate reader (TECAN, Austria) at 540 nm. This experiment was performed in triplicate determination each time (18,19).

## Result and discussion

**Scheme 1:**
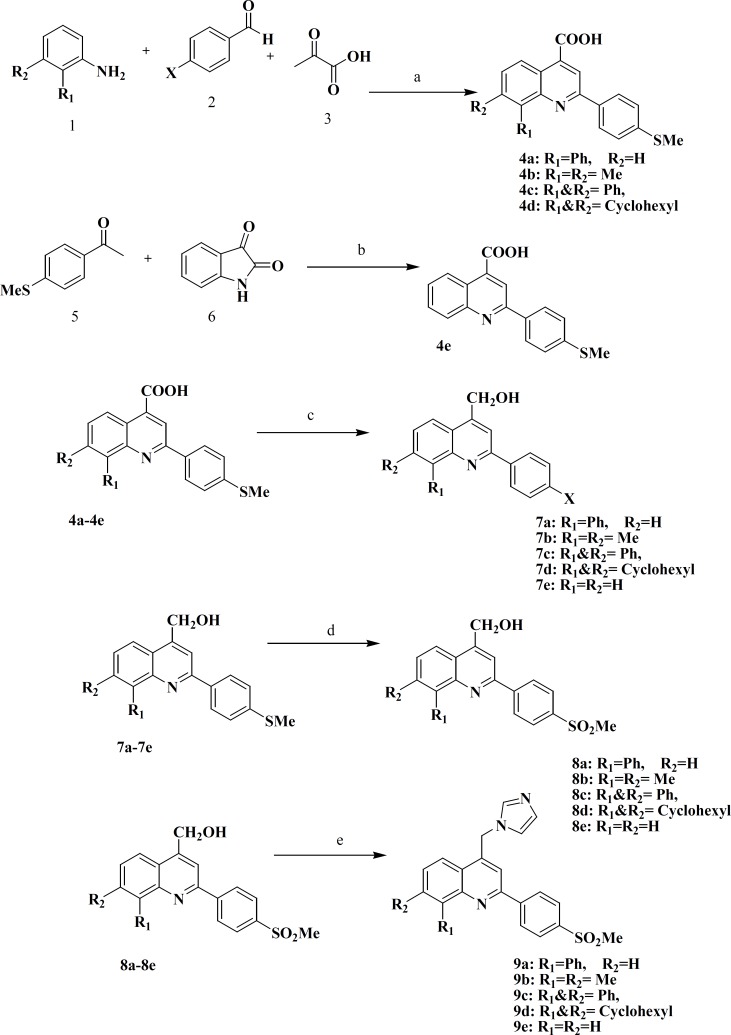
Reagents and conditions: (a) ethanol, reflux, 1-5 h (b) ethanol/KOH, reflux, 48 h (c) LiAlH_4_/THF, 2 h (d) oxone/THF, 2-5 h (e) CDI/NMP, 170°C, 20 h

The synthesis of target compounds was carried out according to Scheme 1. All the synthesized compounds were assayed for their COX-2 inhibitoryandalso cytotoxic activities against breast cancer cell lines (MCF7 and T47D). The results are presented in Table 2.

SAR data (IC_50_ values) acquired by determination of the *in-vitro* ability of the title compoundsto inhibit the COX-1 and COX-2 isozymes showed thatthe COX inhibition was sensitive to the lipophilic nature of substituents.As shown in Table 1, our results showed that the increase oflipophilic properties of substituents on the C-7 and C-8 quinoline ring increased COX-2 inhibitory potency and selectivity. The relative COX-2 potency, and COX-2 selectivity profiles for the 4-imidazolylmethylquinoline derivatives, with respect to the C-7 and C-8 substituents was9d > 9c > 9a > 9b >9e. However, among the 4-imidazolylmethylquinoline derivatives,compound 9d possessing an unsaturated cyclohexyl ring attachedto C-7 and C-8 quinoline ring exhibited highest COX-2 inhibitorypotency and selectivity (COX-2 IC_50_ = 0.063 µM; SI =547.6) thatwasas potent as the reference drug celecoxib and more selective COX-2 inhibitor than celecoxib (COX-2IC_50_ = 0.060 µM; SI = 405).

**Table 1:** *In-vitro *COX-1 and COX-2 enzyme inhibition assay data for 4-(Imidazolylmethyl)-2-(4- methylsulfony phenyl)-Quinolinederivatives(9a-9e).

**Compound**	**R**_1_	**R**_2_	**COX-1 IC**_50_ a **(µM)**	**COX-2 IC**_50_ a **(µM)**	**Selectivity Index** b
**9d**	Cyclohexyl		34.5	0.063	547.6
**9c**	Phenyl		35.9	0.068	527.9
**9a**	Phenyl	H	30.1	0.071	423.9
**9b**	Me	Me	26.0	0.090	288.9
**9e**	H	H	12.95	0.072	179.9
**Celecoxib**			24.3	0.060	405

a Values are means of two determinations acquired using an ovine COX-1/COX-2 assay kit and the deviation from the mean is < 10% of the mean value.

b *In-vitro* COX-2 selectivity index (COX-1 IC50/COX-2 IC50).

**Table 2:** *In-vitro* cytotoxicity of quinolines (9a-9e).

***Compound***	**R** ^1^	**R** ^2^	**IC**_50_ **(µM)**	***Survival *** a *** (%) (10µM )***		**IC**_50_ **(µM)**	***Survival *** b ***(%) (25µM)***	
			***MCF-7***	***48h 72h***		***T47D***	***48h 72h***	
***9d***	cyclohexyl		*<5*	*4*	*0.7*	25	49.1	26.1
***9a***	Ph	H	*<10*	*38.3*	*26.7*	<25	33.8	25.4
***9c***	Phenyl		*10*	*48.4*	*41.3*	50	75.8	68.9
***9b***	Me	Me	*25*	*61*	*47.2*	50	66.2	69.5
***9e***	H	*H*	*>25*	*67.2*	58.2	25	47.4	47.5
***Doxorubicin***	-	-	*0.25*	*50.2*	*31.7*	0.1	53.2	27.1

a Percent survival of MCF-7 cells following exposure to 10 µM concentration of compounds was determined after 48 h and 72 h exposure using MTT assay.

b Percent survival of T47D cells following exposure to 25 µM concentration of compounds was determined after 48 h and 72h exposure using MTT assay.

SAR data (IC_50_ values)also showed that the COX inhibition was sensitive to the nature of substituents on the C-4 quinoline ring. All of the 4-imidazolylmethylquinoline derivatives were less potent but more selective COX-2 inhibitors than their corresponding 4-carboxyl derivatives. Our molecular modeling studies showed that the carboxyl group can interact with Arg^120^ in COX-2, so replacement of carboxyl group withimidazolylmethyl may decrease COX-2 inhibitory activity. In addition, carboxyl group can also interact with Arg^120^ as a key amino acid in COX-1 enzyme, so 4-imidazolylmethylquinoline derivatives have less affinity to bind to COX-1 than the 4-carboxyl derivatives and as a consequence are more selective COX-2 inhibitors.

**Figure 2:**
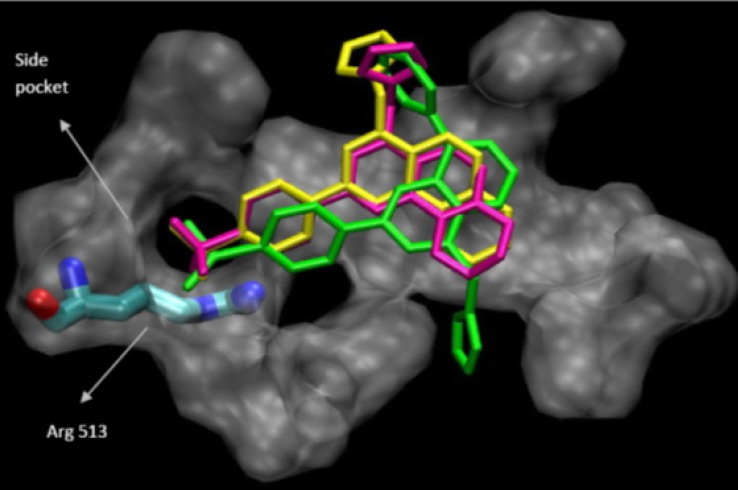
Docking 9a (in green), 9c (in yellow) and 9d (in pink) in the active site of murine COX-2.

The binding interactions of the three most potent and selective COX-2 inhibitor compound (9a, 9c and 9d) within the COX-2 binding site were investigated. They all were docked well in the COX-2 binding site. The most stable enzyme-ligand complex of (9a, 9c and 9d)whichpossessing a MeSO_2_ COX-2 pharmacophore at *para* position of C-2 phenyl ring within the COX-2 binding site (Figure 2) shows that the *p*-MeSO_2_-phenyl moiety is oriented towards the COX-2 secondary pocket (Val^523^, Phe^518^ and Arg^513^). These observations together with experimental results provide a good explanation for design of potent and selective COX-2 inhibitors possessing 4-((1*H*-imidazol-1-yl)methyl)-2-(4-methylsulfonylphenyl)quinoline framework.

The cytotoxicity of quinolines9a-e against human breast cancer MCF-7 and T47D cells by MTT assayafter 2 and 3 days of exposure was also evaluated. After some initial evaluations concentrations of 10 and 25 μM of quinolines were used for evaluation and comparison of cytotoxicity of these compounds with doxorubicin at concentration of 250 nM against MCF-7 cells and concentrations of 25 and 50 μM of quinolines were used for evaluation and comparison of cytotoxicity of these compounds with doxorubicinat concentration of 100 nM against T47D cells. Results for each compound as the percentage of growth of the treated cells in comparison to untreated cells are shown in Table 1. The most active compound against MCF-7 cells was9d, which exhibited 4% of survival (after 2 days exposure) (IC_50_< 5μM). The IC_50_ of 9a was less than 10 μM and the IC_50_ of9c was 10 μM but the IC_50_of 9b and 9e were more than 10μMwhichwere the least potent compounds. The most potent compound against T47D cells was9a which exhibited 33.8% of survival (after 2 days exposure) (IC_50_ ≤ 25μM). Compounds 9c and 9e were the least potent compounds with IC_50_ = 50μM. The calculated IC_50_ values of all tested compounds after two days exposure showed that the order of the cytotoxicity against MCF-7 cells were 9d>9a >9c >9b = 9e and in T47D cells were 9a >9d>9e>9b = 9c. These data showed that the increase of lipophilic properties of substituents on the C-7 and C-8 quinoline ring increased their cytotoxicity on MCF-7. This may be due to their ability to penetrate the cell membrane or their ability to inhibit or suppress some factors and enzymes such as COX-2.As our result showed that the order of the cytotoxicity of quinolines 9a-9eagainst MCF-7 cells were the same as that of their COX-2 inhibitory so one of their cytotoxic mechanism of these compoundsmay be through their COX-2 inhibitory activity. In addition, thesequinolines9a-9e were more cytotoxic against MCF-7 cells in comparison with those of T47D. So this may be due to their different mechanism of action in these two breast cancer cellssuch as more anti-aromatase activity in MCF-7 cells in comparison with T47D cells which express aromatase mRNA less than MCF-7 cells.
